# Prediction of injury localization in preoperative patients with gastrointestinal perforation: a multiomics model analysis

**DOI:** 10.1186/s12876-023-03092-9

**Published:** 2024-01-02

**Authors:** Pingxia Lu, Yue Luo, Ziling Ying, Junrong Zhang, Xiaoxian Tu, Lihong Chen, Xianqiang Chen, Yingping Cao, Zhengyuan Huang

**Affiliations:** 1 Department of Laboratory Medicine, Fujian Medical University Union Hospital, 29 Xinquan Road, Fuzhou, 350001 China; 2 Fujian Medical University, No.1 Xuefu bei Road, Fuzhou, Fujian Province 350122 China; 3 Department of Emergency Surgery, Fujian Medical University Union Hospital, No.29 Xin quan Road, Fuzhou, 350001 Fujian Province China; 4 Department of Medical records management room, Fujian Medical University Union Hospital, 29 Xinquan Road, Fuzhou, 350001 China; 5 Department of Radiology, Fujian Medical University Union Hospital, 29 Xinquan Road, Fuzhou, 350001 China

**Keywords:** Gastrointestinal perforation, Perforation localization, Multiomics model, Laboratory parameters, Radiomics

## Abstract

**Background:**

The location of gastrointestinal perforation is essential for severity evaluation and optimizing the treatment approach. We aimed to retrospectively analyze the clinical characteristics, laboratory parameters, and imaging features of patients with gastrointestinal perforation and construct a predictive model to distinguish the location of upper and lower gastrointestinal perforation.

**Methods:**

A total of 367 patients with gastrointestinal perforation admitted to the department of emergency surgery in Fujian Medical University Union Hospital between March 2014 and December 2020 were collected. Patients were randomly divided into training set and test set in a ratio of 7:3 to establish and verify the prediction model by logistic regression. The receiver operating characteristic curve, calibration map, and clinical decision curve were used to evaluate the discrimination, calibration, and clinical applicability of the prediction model, respectively. The multiomics model was validated by stratification analysis in the prediction of severity and prognosis of patients with gastrointestinal perforation.

**Results:**

The following variables were identified as independent predictors in lower gastrointestinal perforation: monocyte absolute value, mean platelet volume, albumin, fibrinogen, pain duration, rebound tenderness, free air in peritoneal cavity by univariate logistic regression analysis and stepwise regression analysis. The area under the receiver operating characteristic curve of the prediction model was 0.886 (95% confidence interval, 0.840–0.933). The calibration curve shows that the prediction accuracy and the calibration ability of the prediction model are effective. Meanwhile, the decision curve results show that the net benefits of the training and test sets are greater than those of the two extreme models as the threshold probability is 20–100%. The multiomics model score can be calculated via nomogram. The higher the stratification of risk score array, the higher the number of transferred patients who were admitted to the intensive care unit (*P* < 0.001).

**Conclusion:**

The developed multiomics model including monocyte absolute value, mean platelet volume, albumin, fibrinogen, pain duration, rebound tenderness, and free air in the peritoneal cavity has good discrimination and calibration. This model can assist surgeons in distinguishing between upper and lower gastrointestinal perforation and to assess the severity of the condition.

## Introduction

Gastrointestinal perforation (GIP) is one of the most common acute abdominal diseases in general surgery. Untreated GIP may lead to life-threatening complications, including acute diffuse peritonitis, sepsis, and septic shock [1–4]. Clinically, GIP above the Treitz ligament is defined as upper gastrointestinal perforation (uGIP), and the lesion distal to the Treitz ligament is defined as lower gastrointestinal perforation (lGIP). Causes are diverse, gastroduodenal ulcer and gastrointestinal tumors commonly cause uGIP, whereas lGIP is often associated with intestinal tumors, Crohn’s disease, ulcerative colitis, and intestinal diverticulosis [5]. Therefore, it’s important to identify the perforation site.

Currently, imaging technologies, such as abdominal plain film, computed tomography (CT), ultrasound, and magnetic resonance imaging (MRI), alongside patient history and physical examination, are vital for GIP diagnosis. While abdominal plain film offers limited information with a 50–70% diagnostic accuracy, CT’s accuracy reaches 90%, despite requiring higher radiation doses [6]. Ultrasound sensitivity and specificity depend on operator expertise and are susceptible to gas interference. Although MRI lacks ionizing radiation, its application in GIP remains infrequent due to time constraints [7]. Traditional laboratory markers, like procalcitonin (PCT) and C-reactive protein (CRP), reflect inflammation levels, aiding in prognosis differentiation between uGIP and lGIP [8, 9]. Those studies suggest that the etiology, complications, and degree of inflammation burden vary in different perforation sites, which may be reflected by the corresponding levels of laboratory markers. In this study, the integration of clinical features, laboratory markers, and CT imaging was used to construct an effective multi-omics prediction model for the identification of GIP sites.

## Methods

### Patients population

**Fig. 1:**
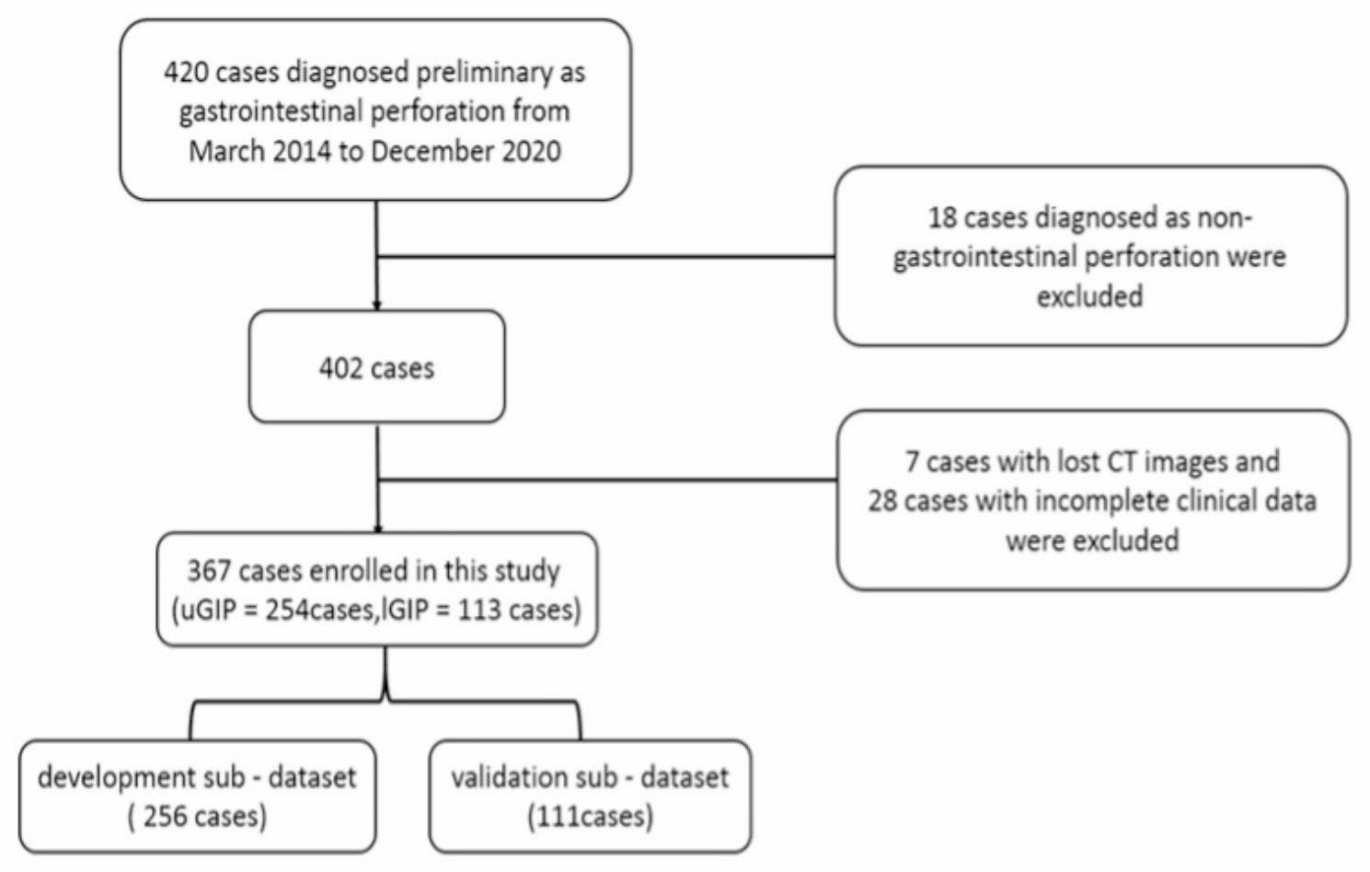
The workflow for the inclusion of the patients

A total of 420 patients who were preliminary diagnosed with GIP between March 2014 and December 2020 at the Department of Emergency Surgery of Union Hospital Affiliated to Fujian Medical University were selected. The 18 patients who were diagnosed as non-GIP, seven patients with missing CT images, and 28 patients with incomplete clinical data were excluded. Finally, a total of 367 patients were recruited in this study, where 254 patients had uGIP and 113 patients had lGIP (Fig. 1). Accordingly, the 367 patients were randomly divided into two groups in a ratio of 7:3, with 256 patients in the training set and 111 cases in the test set. The study was approved by the Institutional Review Board of Fujian Medical University Union Hospital (FJMUUH), and all participants provided written informed consent prior to undergoing the procedures.

### Data collection

General clinical data were collected, including perforation site, gender, age, duration of abdominal pain, tenderness, rebound tenderness, abdominal muscle tension, bowel sounds, hospitalization cost, the length of hospital stay, and whether to be transferred to intensive care unit (ICU). The criteria for ICU admission were both signs of peritonitis (abdominal muscle tension, tenderness, rebound tenderness) and qSOFA (quick Sequential Organ Failure Assessment) score ≥ 2. Laboratory findings, including neutrophil absolute value (Neu), lymphocyte absolute value (Lym), monocyte absolute value (MC), hemoglobin (Hb), mean platelet volume (MPV), total bilirubin (TBIL), albumin (ALB), D-dimer (DDI), fibrinogen (FIB), were recorded as continuous variables from the database.

### CT findings

**Fig. 2:**
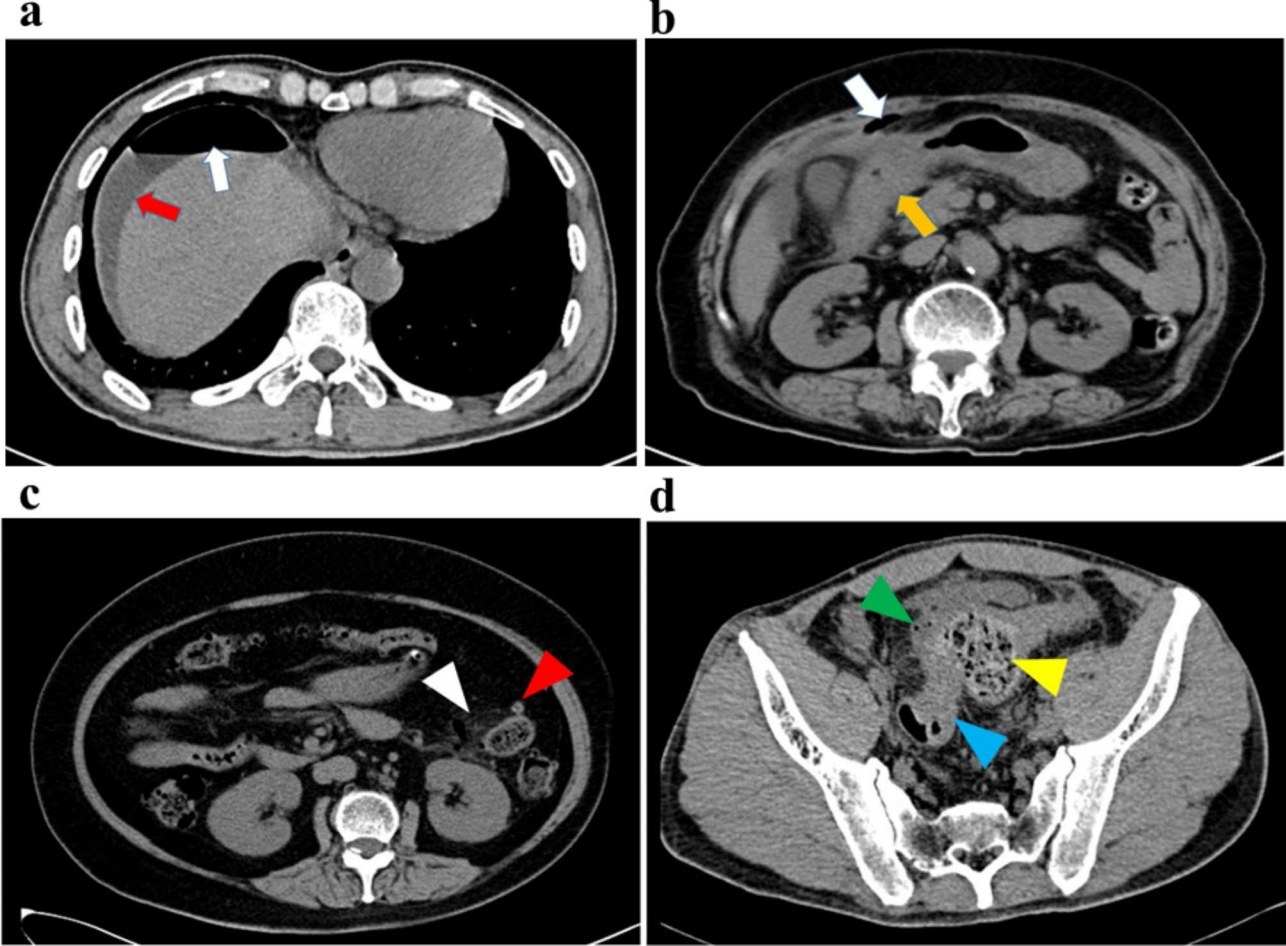
Abdominal CT for the patients (**a**) a 49-year-old man with perforation of the duodenal ulcer. Abdominal CT showed signs of subphrenic free gas (white arrow), perihepatic effusion (red arrow). (**b**) a 78-year-old woman with perforation of the duodenal ulcer. Abdominal CT showed signs of subphrenic free gas (white arrow), duodenal wall edema (orange arrow). (**c**) a 68-year-old woman with perforated diverticula of the descending colon. Abdominal CT showed inflammation and edema of tissues surrounding the descending colon (white triangle), diverticular lesion in intestinal wall (red triangle). (**d**) a 50-year-old man with perforation of the sigmoid carcinoma. Abdominal CT showed local bowel wall edema, stingy bubble (green triangle), carcinomatous lesion thickened, lumen stenosis (blue triangle), fecal retention proximal to the lesion (yellow triangle)

The CT scans were performed in all the patients before receiving any treatment. The characteristics of CT images were classified according to the presence free air (FA) above (UFA) and below the transverse mesocolon (LFA), ascites, and edema of the gastrointestinal wall (Fig. 2). Two senior radiologists independently analyzed the CT scans. When the analyses of each film were consistent, the conclusion was confirmed. Any discrepancy in the analysis was discussed and determined with an experienced general surgeon with more than ten years of experience in acute abdominal surgeries.

### Statistical analyses

Continuous variables are presented as mean ± standard deviation (SD). Student’s *t*-test was used for comparisons between independent samples with normal distribution. Meanwhile, Mann-Whitney test was used for samples that do not have normal distribution, and the variables are expressed as median and interquartile range (IQR). Categorical variables are expressed as rates and were analyzed using Chi-square test. Univariate logistic regression analysis was used to screen the primary candidate factors (*P* < 0.05) and the cut-off value of the receiver operating characteristic curve (ROC) was selected and converted into dichotomous variables for multivariate logistic regression analysis (*P* < 0.05). Finally, stepwise regression was used to determine the prediction model according to Akaike Information criterion (AIC), and a nomogram was created. The complete subjects were randomly assigned to the training and test sets in a 7:3 ratio. The performance of the prediction model was evaluated respectively using discrimination, calibration, and clinical net benefit in the training and test sets. SPSS (version22.0) and R (version4.12) software were used for the statistical analyses.

## Results

### Comparison between the basic demographics and laboratory parameters in the training and test sets

There was no significant difference with respect to the demographics between the proportion of patients with uGIP and lGIP in the training and test sets (*P* > 0.05). Except for Hb levels, the other laboratory indices showed no statistical significance between the two groups (*P* > 0.05). Accordingly, there is a balance between the samples of the training and test sets (Table 1).

**Table 1:** Comparison of the basic demographics and laboratory parameters in the training and test sub-datasets of patients with gastrointestinal perforation

	Subjects (n = 367)	Training dataset (n = 256)	Test dataset (n = 111)	*P*-value
**Perforation site(%)**				0.937
Upper-GIP	254(69.21)	178(69.53)	76(68.47)	
Lower-GIP	113(30.79)	78(30.47)	35(31.53)	
**Gender n(%)**				0.491
Male	294(80.11)	208(81.25)	86(77.48)	
Female	73(19.89)	48(18.75)	25(22.52)	
**Age (year)**	56.00[40.00,70.00]	56.00[40.00,69.25]	56.00[39.50,71.00]	0.971
**Laboratory parameters**				
TBIL(μmol/L)	18.60[13.75,4.70]	18.60[13.75,4.63]	17.60[13.80,24.85]	0.645
ALB(g/L)	38.40[33.30, 2.30]	38.70[33.95,42.63]	37.70[31.50,1.60]	0.201
DDI(mg/L)	1.66[0.85,3.51]	1.66[0.88,3.26]	1.65[0.83,3.90]	0.546
FIB(g/L)	3.88[3.15,5.14]	3.84[3.15,5.26]	4.01[3.15,4.97]	0.989
Neu(10^9/L)	11.03[7.23,14.73]	10.67[6.92,14.50]	11.75[8.56,15.26]	0.081
Lym(10^9/L)	0.80[0.57,1.21]	0.78[0.57,1.20]	0.83[0.59,1.26]	0.337
MC(10^9/L)	0.61[0.40,0.87]	0.59[0.39, 0.86]	0.67[0.42,0.96]	0.104
MPV(fL)	9.90[9.10,10.80]	9.90[9.10,10.70]	9.80[9.20,10.95]	0.571
Hb(g/L)	140.00 [124.50,150.00]	142.50 [127.75,151.00]	136.00 [120.50, 149.00]	0.032
**Pain duration(%)**				0.508
Yes	288(78.47)	198(77.34)	90(81.08)	
No	79(21.53)	58(22.66)	21(18.92)	
**Tenderness(%)**				0.778
None	9(2.45)	6(2.34)	3(2.70)	
complete abdomen	240(65.40)	163(63.67)	77(69.37)	
upper abdomen	71(19.35)	54(21.09)	17(15.32)	
lower abdomen	30(8.17)	21(8.20)	9(8.11)	
Others	17(4.63)	12(4.69)	5(4.50)	
**Rebound tenderness (%)**				0.477
None	60(16.35)	38(14.84)	22(19.82)	
complete abdomen	186(50.68)	129(50.39)	57(51.35)	
upper abdomen	60(16.35)	46(17.97)	14(12.61)	
lower abdomen	28(7.63)	18(7.03)	10(9.01)	
Others	33(8.99)	25(9.77)	8(7.21)	
**Bowel sounds(%)**				0.397
Absent	13(3.54)	10(3.91)	3(2.70)	
Hypoactive	266(72.48)	186(72.66)	80(72.07)	
Normal	83(22.62)	55(21.48)	28(25.23)	
Hyperactive	5(1.36)	5(1.95)	0(0.00)	
**Tension of abdominal muscle (%)**				0.785
Soft	59(16.08)	40(15.62)	19(17.12)	
slightly tense	126(34.33)	86(33.59)	40(36.04)	
Tense	182(49.59)	130(50.78)	52(46.85)	
**Local seroperitoneum(%)**				0.092
Yes	198(53.95)	146(57.03)	52(46.85)	
No	169(46.05)	110(42.97)	59(53.15)	
**FA(%)**				0.137
no/LFA	48(13.08)	30(11.72)	18(16.21)	
UFA	207(56.40)	143(55.86)	64(57.66)	
UFA + LFA	112(30.52)	83(32.42)	29(26.13)	
**Edema of bowel wall (%)**				0.504
Yes	187(50.95)	127(49.61)	60(54.05)	
No	180(49.05)	129(50.39)	51(45.95)	

GIP: gastrointestinal perforation, Neu: neutrophil absolute value, Lym: lymphocyte absolute value, MC: monocyte absolute value, Hb: hemoglobin, MPV: mean platelet volume, TBIL: total bilirubin, ALB: albumin, DDI: D-dimer, FIB: fibrinogen, FA: free air in peritoneal cavity, UFA: upper FA, LFA: lower FA.

### Univariate logistic regression analysis and data transformation

**Fig. 3:**
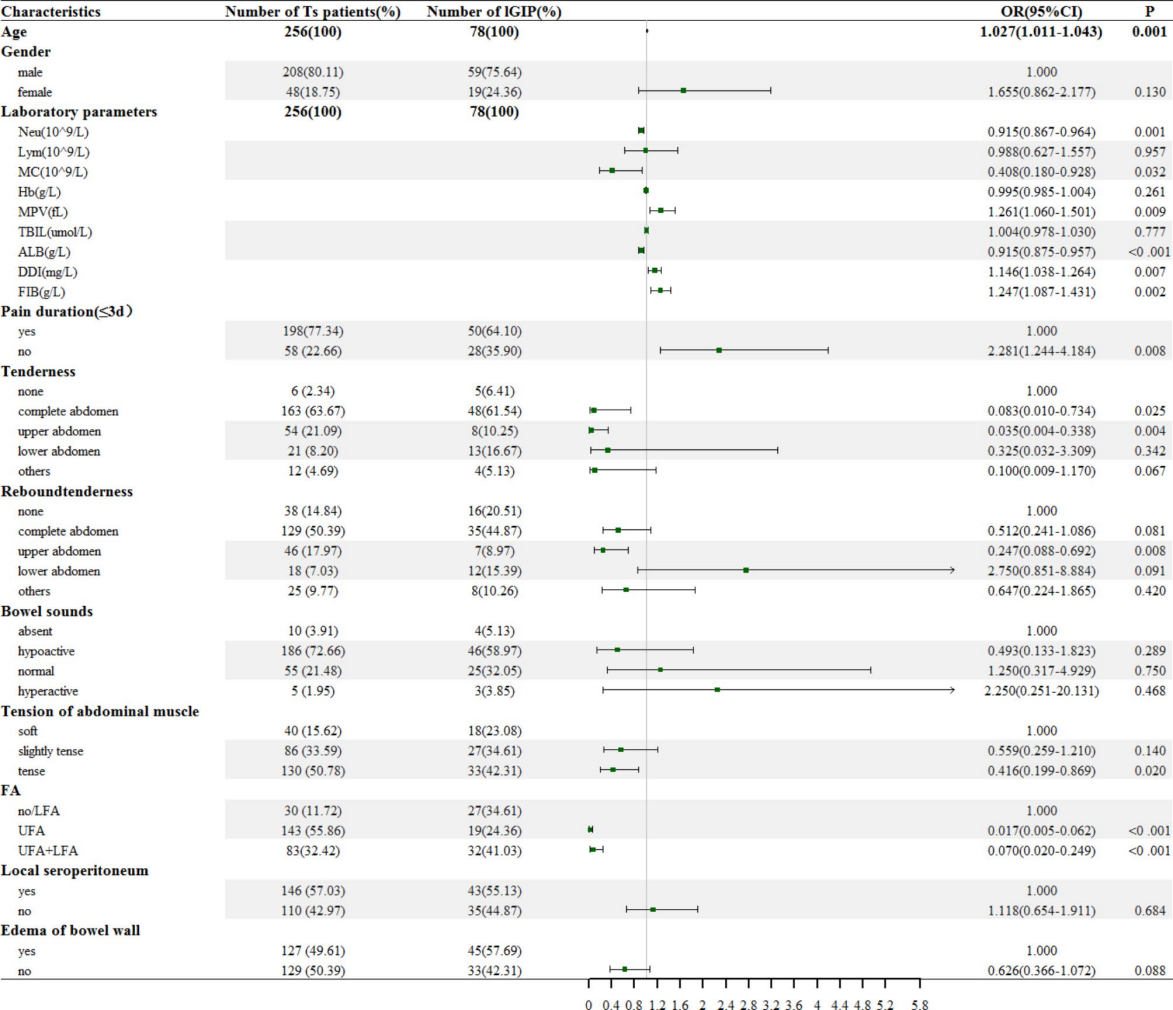
The forest plot of univariate logistic regression analysis for the preoperative prediction of localization in patients with gastrointestinal perforation. Ts patient: patient of training set

A total of 12 candidate variables were preliminarily screened out from the 19 collected variables, including baseline characteristics, laboratory indicators, and imaging characteristics of the patients in the training set (Ts patients), by univariate logistic regression analysis (Fig. 3). The baseline characteristic predictive variables included age (*P* < 0.001), pain duration (P_no_=0.008), tenderness (P_upper abdomen_=0.004), rebound tenderness (P_complete abdomen_=0.008), and tension of abdominal muscle (P_tense_=0.020). The predictive variables of the laboratory indicators included Neu (*P* = 0.001), MC (*P* = 0.032), MPV (*P* = 0.009), ALB (*P* = 0.001), DDI (*P* = 0.007), and FIB (*P* = 0.002). Meanwhile, the imaging characteristic variables included FA (P_UFA_<0.001; P_UFA+LFA_<0.001). The six aforementioned continuous variables, except for age, were converted into binary variables (Table 2).

**Table 2:** Assignment table of the multivariate logistic regression analysis of the preoperative perforation location in patients with gastrointestinal perforation

Variables	Value assignment
Neu(10^9/L)	< 9.805(10^9/L) = 0, ≥ 9.805(10^9/L) = 1
MC(10^9/L)	< 0.555(10^9/L) = 0, ≥ 0.555(10^9/L) = 1
MPV(fL)	< 11.650fL = 0, ≥ 11.650fL = 1
ALB(g/L)	< 39.950 g/L = 0, ≥ 39.950 g/L = 1
DDI(g/L)	< 2.210 g/L = 0, ≥ 2.210 g/L = 1
FIB(g/L)	< 4.920 g/L = 0, ≥ 4.920 g/L = 1
Duration(≤ 3d)	no = 0, Yes = 1
Tenderness(%)	none = 0, complete abdomen = 1, upper abdomen = 2, lower abdomen = 3, others = 4
Rebound tenderness(%)	none = 0, complete abdomen = 1, upper abdomen = 2, lower abdomen = 3, others = 4
Tension of abdominal muscle(%)	soft = 0, slightly tense = 1, tense = 2
FA	no/LFA = 0, UFA = 1, UFA + LFA = 2
Location of perforation	upper = 0, lower = 1

Neu: neutrophil absolute value, MC: monocyte absolute value, Hb: hemoglobin, MPV: mean platelet volume, ALB: albumin, DDI: D-dimer, FIB: fibrinogen, FA: free air in peritoneal cavity, UFA: upper FA, LFA: lower FA.

### Multivariate logistic regression and prediction model construction

**Fig. 4:**
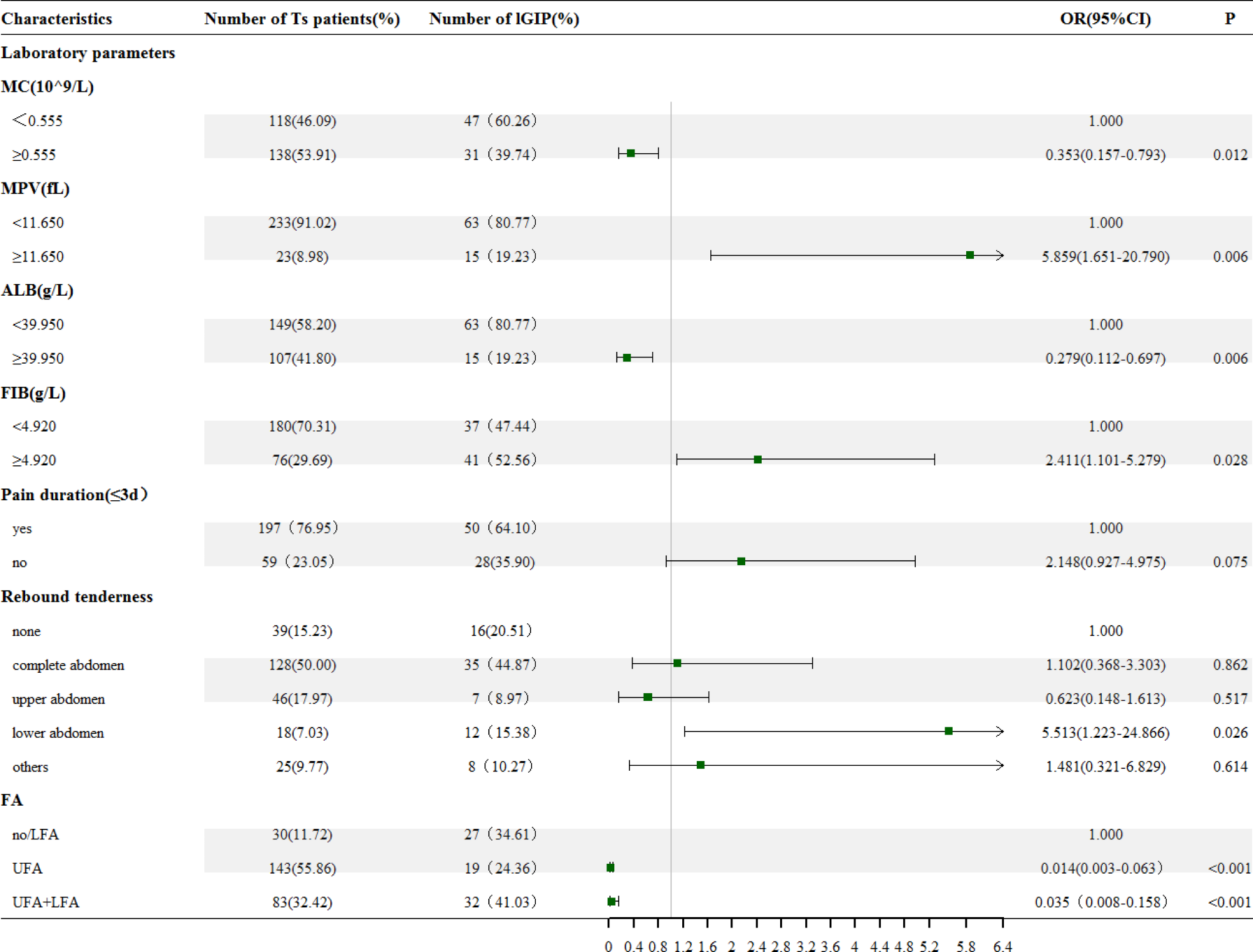
The forest plot of the multivariate logistic regression analysis for prediction. Ts patient: patient of training set

**Fig. 5:**
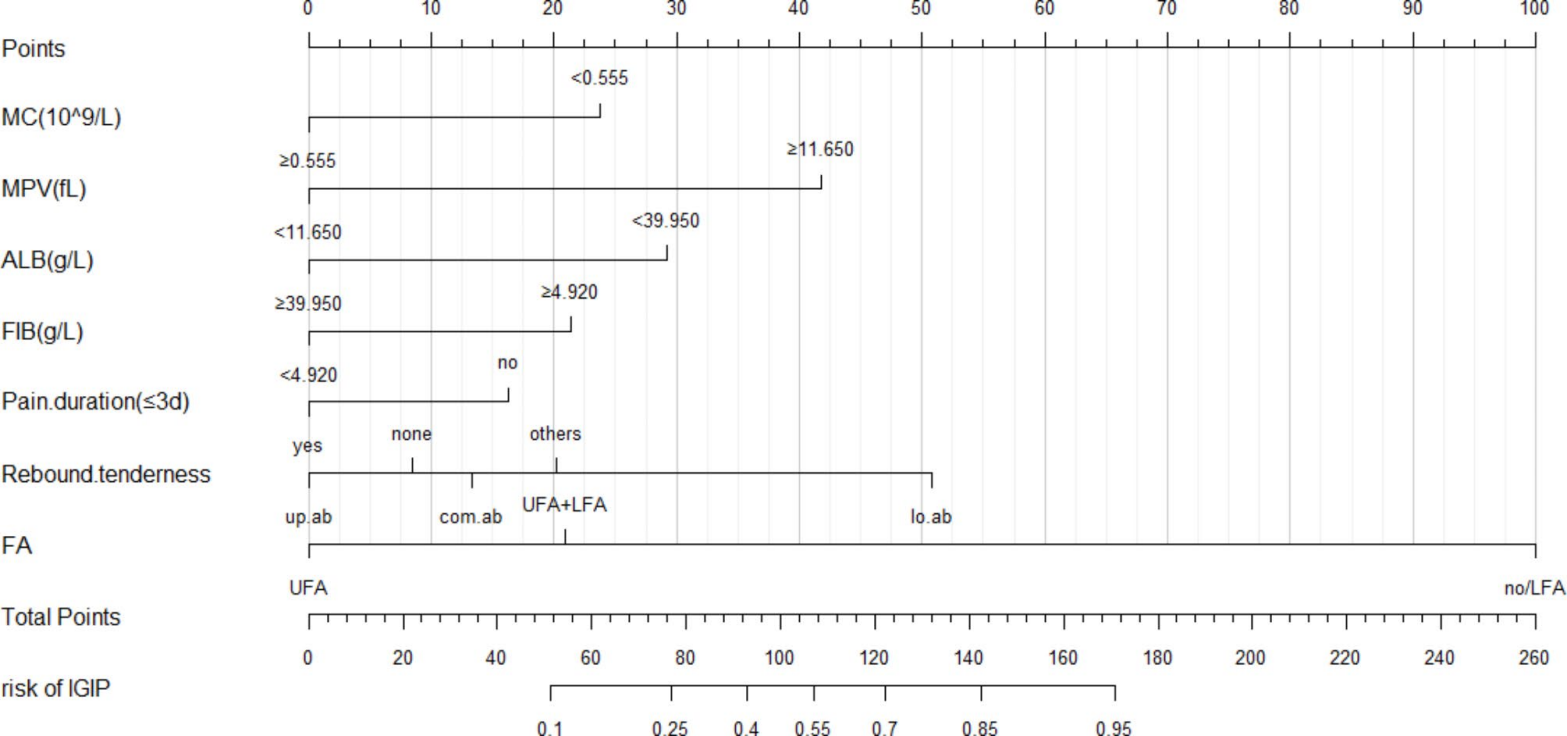
The nomogram of the prediction model

Multivariate logistic regression prediction model was constructed by screening the variables as independent variables, except for the uGIP and lGIP which were screened as dependent variables (Table 2). The results revealed that the regression model with the minimum AIC value of 212.01 was the most feasible model. The seven variables included (OR_lower abdomen_=5.513), levels of MC (OR = 0.353), MPV (OR = 5.859; ), ALB (OR = 0.279), and FIB (OR = 2.411), pain duration (OR_no_=2.148), rebound tenderness and the imaging characteristic variables UFA (OR = 0.0141), and UFA + LFA(OR = 0.035) (Fig. 4). The nomogram was plotted according to the retrieved predicted variables (Fig. 5).

### Evaluation of predictive model

**Fig. 6:**
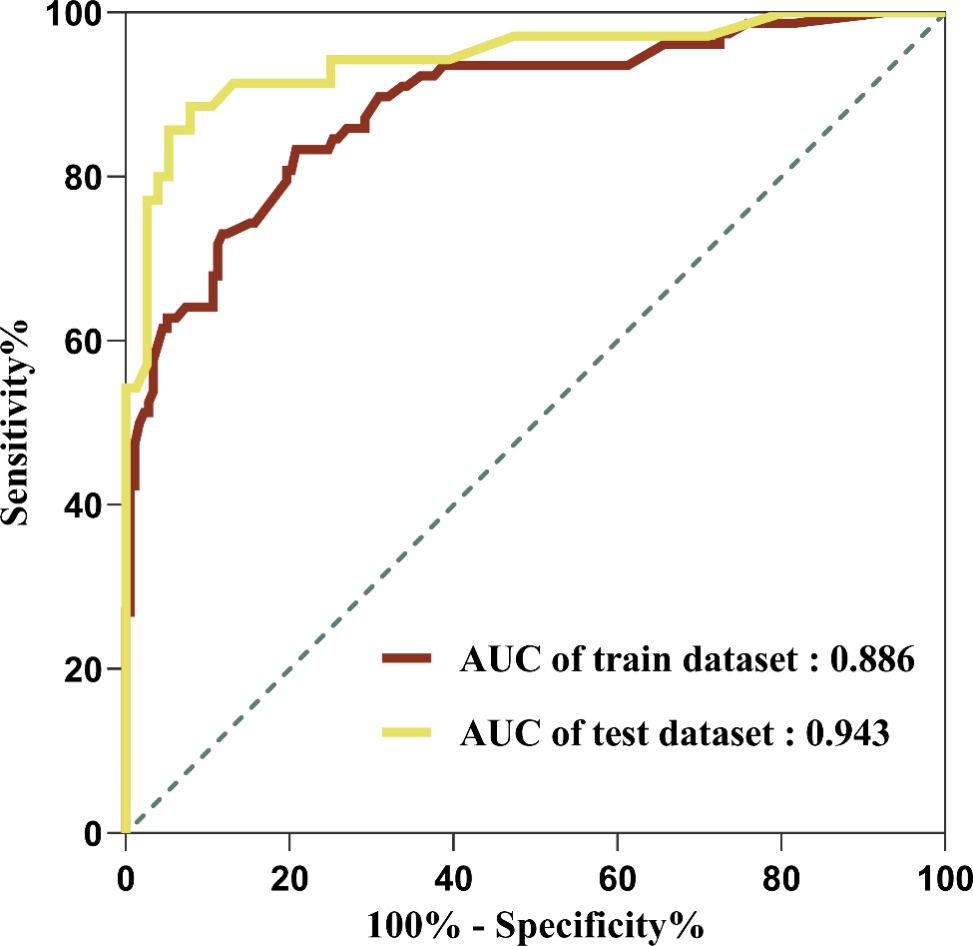
The ROC of the prediction model

**Fig. 7:**
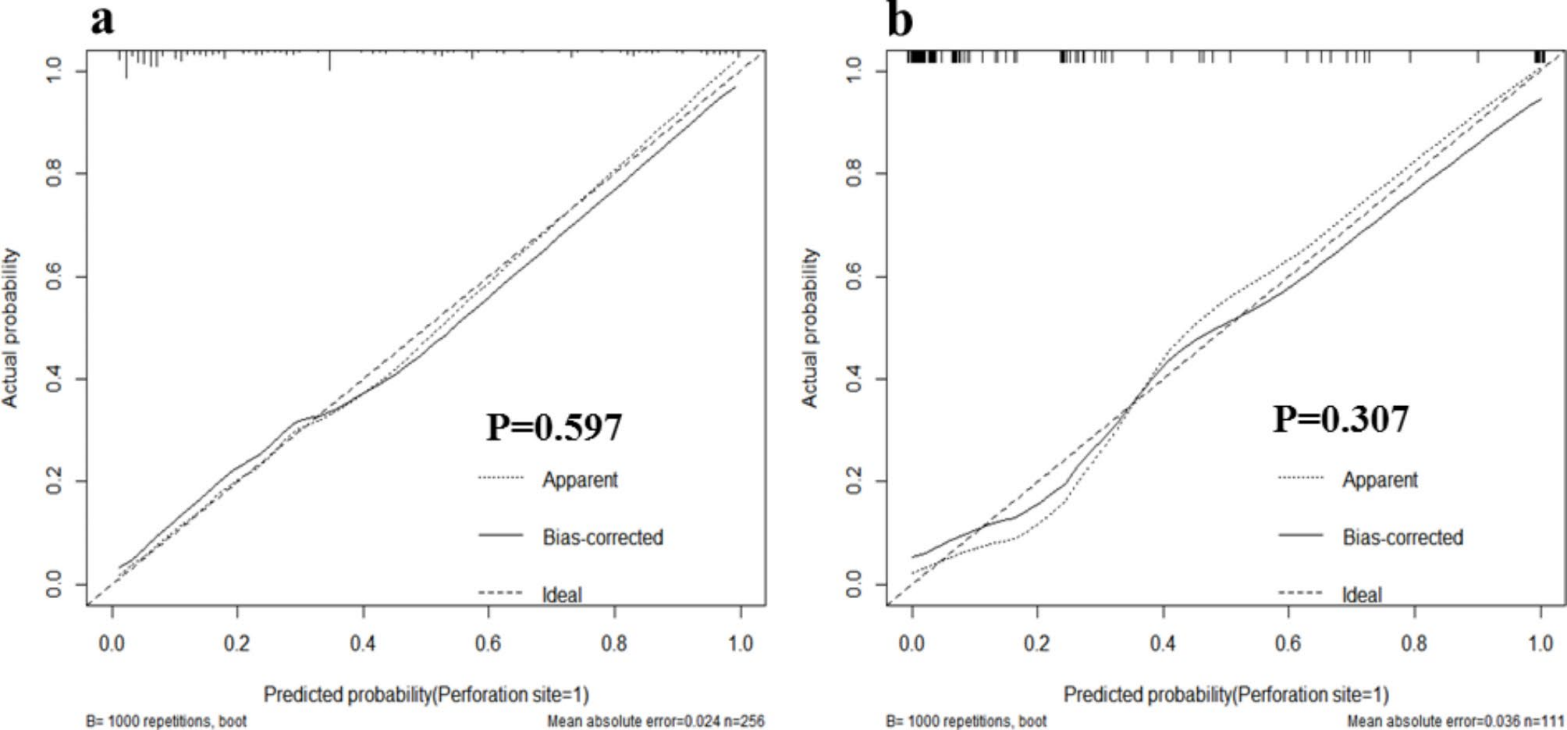
The calibration curve of the prediction model in the training dataset and test dataset. (**a**) training dataset and (**b**) test dataset

The area under the ROC curve (AUC) of the prediction models of the training and test sets were 0.886 (95% CI, 0.840–0.933) and 0.943 (95% CI, 0.891–0.995; *P* = 0.273), respectively (Fig. 6). The two prediction models have good discriminative abilities. The calibration curve of the prediction model showed that the predicted risk of the model was highly consistent with the actual risk of the disease, and the correction ability of the model was also effective (Fig. 7).

### Clinical application of predictive model

**Fig. 8:**
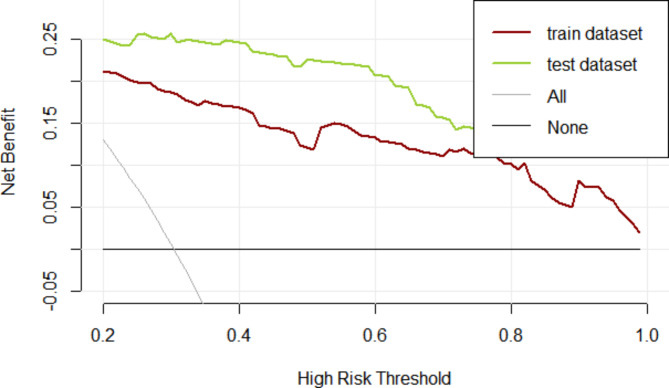
The DCA of the prediction model in the training dataset and test dataset

The DCA results of the upper gastrointestinal perforation risk diagram showed that when the threshold probability was within the range of 20–100%, the net benefit of the prediction model was significantly greater than that of the two extreme models (with or without intervention) (Fig. 8).

### Validation of the multiomics models for the prediction of severity and prognosis of GIP

**Fig. 9:**
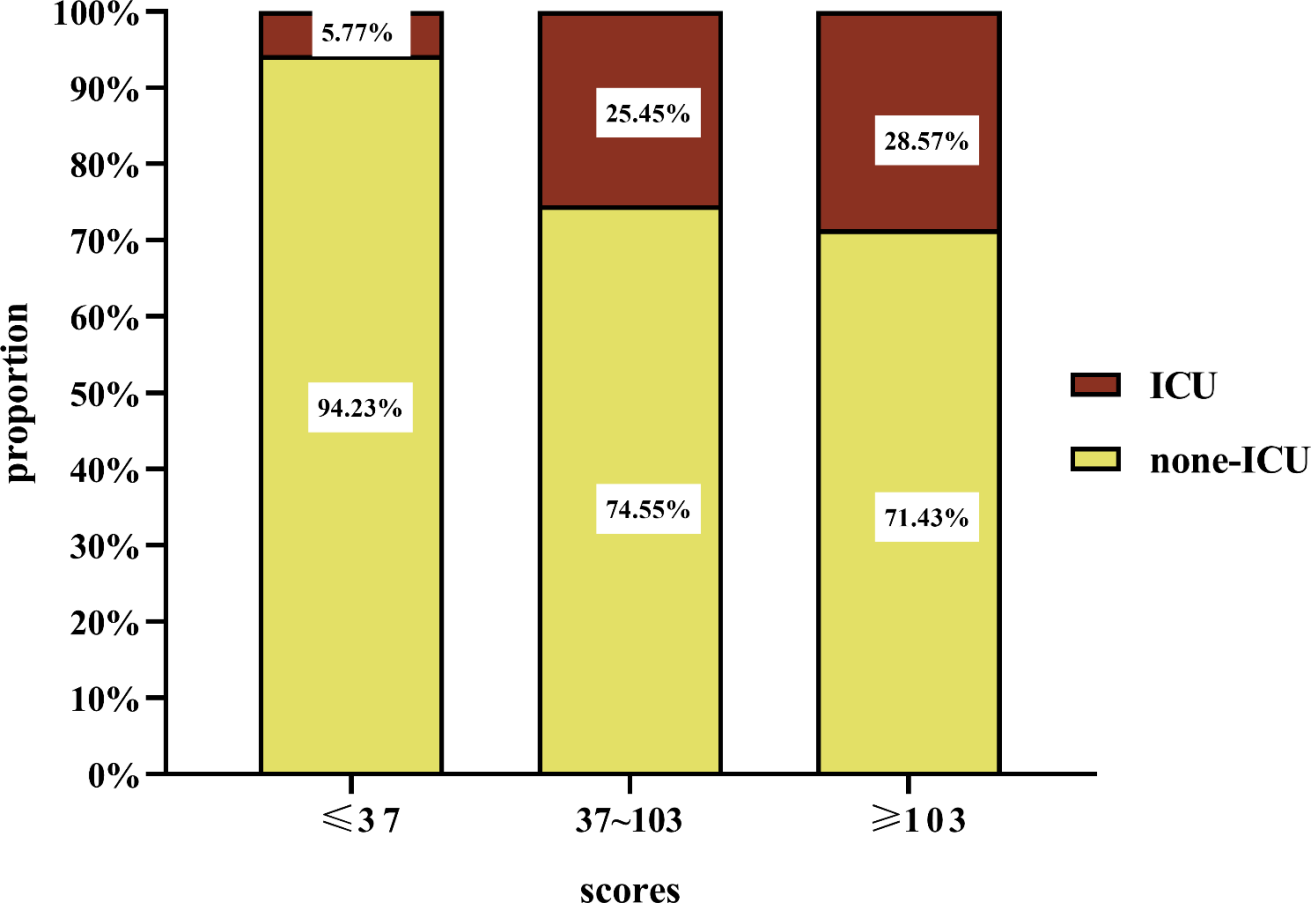
Validation of the multi-omics models for prediction of ICU admission in patients with GIP

**Fig. 10:**
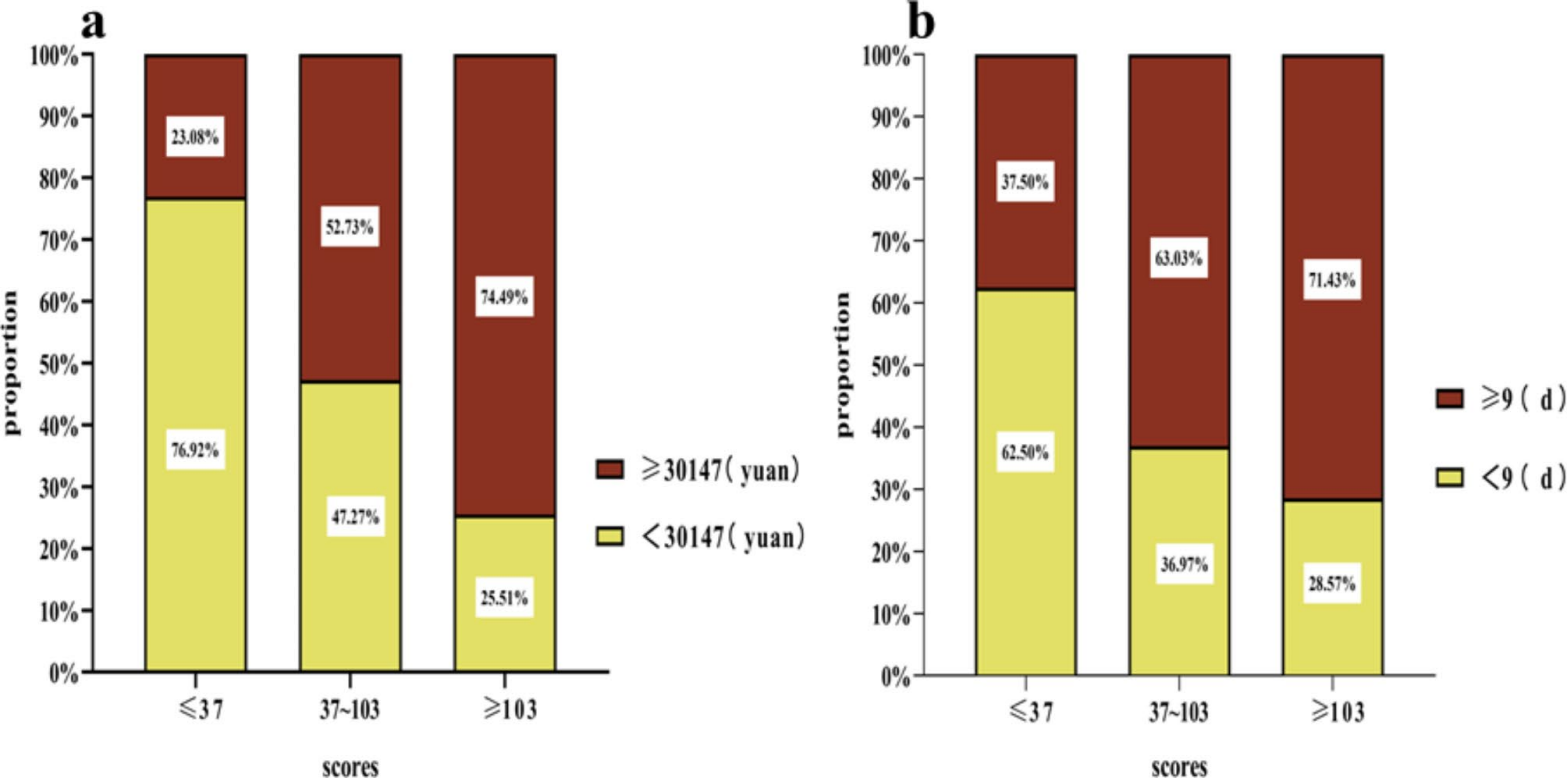
Validation of the multi-omics models for prediction of hospitalization costs (**a**) and the length of hospital stay (**b**) in patients with GIP

The patients were further divided into three groups based on the fourth quartile of the multiomics model scores; a low-risk group (risk scores ≤ 37; n = 104), a medium-risk group (37 < risk scores ≤ 103; n = 165), and a high-risk group (risk scores > 103, n = 98). The severity of the GIP condition was evaluated based on whether or not the patient was admitted to the intensive care unit (ICU). The number of patients admitted to the ICU was the lowest in the low-risk group (5.77%) and the highest in the high-risk group (28.57%). The higher the score, the more patients that were admitted to the ICU (*P* < 0.001; Fig. 9). Accordingly, the length of hospital stay and hospitalization costs in the high-risk group with high score array also showed a significant increase compared to the low-risk group (*P* < 0.001; Fig. 10).

## Discussion

GIP is one of the common surgical acute abdomen-related conditions, which can be fatal in severe cases. It has various etiologies, locations, treatment modalities, and prognoses. The preoperative judgment of the perforation site is very important for the surgical plan. Currently, surgeons mainly rely on the patient history and physical examination combined with imaging techniques to diagnose GIP.

The occurrence of GIP often presents with signs of peritoneal irritation, including abdominal tenderness, rebound pain, and abdominal muscle tension [10, 11]. Chemical peritonitis caused by gastric fluid, bile, and pancreatic fluid entering the peritoneal cavity usually results in significant abdominal pain due to uGIP in the early stage. However, the irritating symptoms of chemical peritonitis caused by lGIP may be non-significant owing to the differences in bowel contents and flora. On the other hand, bacterial peritonitis can be predominant in the early stage [12, 13]. In the univariate regression analysis, the duration, tenderness, rebound tenderness, and tension were significant. Whereas in the multivariate regression, only the duration and rebound tenderness were significant. This may be attributed to the false positive peritoneal stimulation signs in the patients with tension or involuntary contraction of the abdominal muscles, or false negative peritoneal stimulation signs due to the reduced reactivity caused by increasing age, infirmities, poisoning, coma, sedation, and analgesia [11]. CT has been widely used in the diagnosis of GIP in recent years because of its advantages of high-density and spatial resolution compared with abdominal radiographs, among which the distribution of free air outside the lumen is the most important sign [14, 15]. Due to the anatomical characteristics of the omentum, mesangium, ligaments, and organs in the abdominal cavity, the free gas caused by GIP in different sites has a certain distribution rule. However, the specificity and sensitivity of CT imaging were different. Celik et al. reported that the free air around the portal vein, liver, and stomach is of great significance in gastroduodenal perforation with sensitivities of 86.4%, 54.2%, and 84.7%, and specificities of 40.3%, 69.3%, and 48.4%, respectively [16]. Cho et al. also reported that the presence of free air around the portal vein indicates a higher possibility of uGIP [17]. In this study, the transverse mesocolon was used as the boundary. Accordingly, the free gas was classified as above the transverse mesocolon (in the subphrenic space, around the falciform ligament of the liver, around the round ligament, and around the hilar of the liver) or below the transverse mesocolon (around the small mesocolon, ascending colon, sigmoid colon, and mesocolon). The distribution of free gas above and below the transverse mesentery had a significant effect in discriminating the localization of uGIP and lGIP (P_UFA_<0.001 and P_UFA + LFA_<0.001, respetctively), which was similar to the aforementioned results.

The acquisition of laboratory parameters was relatively simple, fast, and inexpensive. In this study, four laboratory indicators, namely MC, MPV, ALB, and FIB, were included in the prediction model.

Monocytes and/or macrophages are important non-specific immune cells in the body. GIP causes severe peritoneal cavity inflammation, leading to the depletion of defense mechanisms such as peritoneal macrophages, neutrophils, and complement aggregation. It also aggravates the inflammatory load and coagulation load of the body [12, 13]. In this study, MC (OR, 0.353; *P* = 0.012) was found to be an important predictor of upper and lower digestive tract perforation, and the prediction model showed that MC level in uGIP is higher than that in lGIP.

MPV is also one of the commonly used indicators of inflammation. Studies have found that the MPV level in the patients with a positive blood culture is higher than that in the patients with a negative blood culture [18, 19], suggesting that the increase in MPV level is a sign that the patients are progressing from having local infection to systemic infection, thus, it can be used to evaluate the severity and prognosis of sepsis. The results of this study show that MPV (OR, 5.859; *P* = 0.006) is one of the prognostic factors for digestive tract perforation, which may be related to systemic inflammation caused by secondary infection. Excessive inflammation causes over-activation of platelets, which in turn results in over-consumption of platelets. Bone marrow produces more platelets as a compensatory mechanism to supplement the over-consumption of platelets. The platelets that are produced by over-activation undergo changes in their morphology and function, which can be reflected by MPV [20].

FIB can be used as a molecular marker for hypercoagulability and thrombosis. Over-activated inflammatory reactions and coagulation disorders will result in a vicious coagulation cycle [21, 22]. Our study showed that lGIP was more likely to occur in the patients with GIP having FIB levels ≥ 4.920 g/L than those having FIB levels < 4.920 g/L. Recently, studies have reported that there are high FIB levels in pancreatic cancer, colorectal cancer, and other gastrointestinal system malignancies [23, 24], suggesting that the baseline FIB level may be high in patients with tumor-induced lGIP.

ALB, a negative acute phase protein produced by the liver, is a traditional nutritional and inflammatory marker. After abdominal infection, the absorption of endotoxin increases, thus stimulating the production of inflammatory mediators such as TNF, IL-1, and IL-6 in liver macrophages and inhibiting the translation of the ALB transcript, ultimately leading to hypoalbuminemia [25]. The results of this study showed that ALB (OR, 0.279; *P* = 0.006) was an important predictor for distinguishing between uGIP and lGIP, and the ALB level in patients with lGIP was lower than that in patients with uGIP. On the one hand, there are differences in the basic nutrition of these patients. The etiology of uGIP is mainly attributed to ulcerative diseases, but rarely cancer [3]. In lGIP, small bowel perforation is often caused by intestinal ischemia or inflammatory bowel Crohn’s disease. Colorectal perforation induced by colorectal cancer and diverticulitis is relatively common [26]. Furthermore, perforation that occurs in colorectal cancer is considered as a late-stage complication, and patients with colorectal cancer mostly have changes in protein metabolism, which are mainly manifested as skeletal muscle atrophy, hypoproteinemia, and other manifestations of cachexia [27, 28]. On the other hand, the pathophysiological mechanism of infection caused by GIP in different sites varies according to the microenvironment and microflora. Gram-positive *cocci* are most frequently detected in patients with uGIP and can play an important pathological role through their virulence factors such as capsular polysaccharide, exotoxin, extracellular enzyme, and adhesin [29]. However, gram-negative *Bacilli* and anaerobic bacteria are often detected in patients with lGIP. They mainly exert their pathological role through their toxic factors such as endotoxin, adhesion, and immunomodulatory molecules [30].

Nomogram has been widely used for the risk prediction and prognosis assessment of malignancies and chronic diseases [31–35]. The complex multi-factor logistic regression prediction model was transformed into a visual graph through analysis and integration, and was used to predict the GIP. The AUC of the training set was 0.886, and that of the test set was 0.943, suggesting that the model had a high ability to distinguish between uGIP and lGIP. Meanwhile, the calibration curves of both the training set and the test set in this study showed good consistency between the predicted probability of the model and the actual probability. As an emerging method of evaluation and prediction, DCA provides decision evaluation by comparing the size of net benefit values [36–39]. The DCA curves of the training and the test set of the prediction model indicate that the prediction value of the model has good validity. Furthermore, the patients were divided into three groups and the severity of the condition of GIP was evaluated based on whether or not the patient was admitted to the ICU. The number of patients who were admitted to the ICU was the least in the lowest scoring group and the highest in the highest scoring group. Moreover, the length of hospital stay and hospitalization costs in the high-risk group also increased significantly with the increasing scores. Thus, more attention should be paid to patients in high-risk groups when applying this model to predict the perforation site. For critical patients with poor basic condition, damage control surgery should be urgently adopted, and staging surgery is also feasible.

There are some shortcomings in this study. First, it is a single-center retrospective study. A multi-center prospective study is needed to evaluate the actual performance of the prediction model. Secondly, the predictive effectiveness of the column graph prediction model needs to be verified using more data, especially in multi-center large-sample cohorts. Third, the model should indicate contraindications and applicable constraints as far as possible. If data for certain types of patients (such as immunosuppressed patients) are not included, the model may not be applicable in this population. Fourth, there is no data on patient complications, mortality, re-hospitalization rate, post-discharge follow-up records, etc., so the model could not provide a reference for patient prognosis.

## Conclusions

A predictive model for preoperative positioning of GIP was constructed. It included seven predictive variables, which are pain duration, rebound tenderness, FA, MC, MPV, ALB, and FIB. It can be used as a useful auxiliary tool for distinguishing between uGIP and lGIP and evaluating the severity of patients.

## Data Availability

The datasets supporting the conclusions of this study are included within the article. Corresponding to Yingping Cao and Zhenghuang Huang when necessary.

## List of abbreviations

AIC: Akaike Information criterion
ALB: Albumin
AUC: Area under the curve
CT: Computed tomography
DDI: D-dimer
FA: Free air in peritoneal cavity
FIB: Fibrinogen
GIP: Gastrointestinal perforation
Hb: Hemoglobin
ICU: Intensive care unit
LGIP: Lower gastrointestinal perforation
Lym: Lymphocyte absolute value
MC: Monocyte absolute value
MPV: Mean platelet volume
MRI: Magnetic resonance imaging
Neu: Neutrophils absolute value
ROC: Receiver operating characteristic
TBIL: Total bilirubin
uGIP: Upper gastrointestinal perforation
